# External quality assessment of Rift Valley fever diagnosis in 17 veterinary laboratories of the Mediterranean and Black Sea regions

**DOI:** 10.1371/journal.pone.0239478

**Published:** 2020-09-28

**Authors:** Elisa Pérez-Ramírez, Cristina Cano-Gómez, Francisco Llorente, Bojan Adzic, Maisa Al Ameer, Igor Djadjovski, Jeanne El Hage, Fatiha El Mellouli, Teufik Goletic, Hermine Hovsepyan, Ilke Karayel-Hacioglu, Jelena Maksimovic Zoric, Selma Mejri, Hassiba Sadaoui, Sayed Hassan Salem, Kurtesh Sherifi, Natela Toklikishvili, Ani Vodica, Federica Monaco, Alejandro Brun, Miguel Ángel Jiménez-Clavero, Jovita Fernández-Pinero

**Affiliations:** 1 Centro de Investigación en Sanidad Animal, Instituto Nacional de Investigación y Tecnología Agraria y Alimentaria (INIA‐CISA), Valdeolmos, Spain; 2 Diagnostic Veterinary Laboratory, Podgorica, Montenegro; 3 Animal Wealth Laboratory Sector, Ministry of Agriculture, Amman, Jordan; 4 Faculty of Veterinary Medicine, Ss. Cyril and Methodius University, Skopje, Republic of North Macedonia; 5 Animal Health Laboratory, Lebanese Agricultural Research Institute, Fanar, Lebanon; 6 Casablanca Regional Research and Analysis Laboratory of National Office of Sanitary Safety and Food Products (ONSSA), Nouaceur, Casablanca, Morocco; 7 Veterinary Faculty, University of Sarajevo, Sarajevo, Bosnia and Herzegovina; 8 Republican Veterinary-Sanitary and Phytosanitary Center of Laboratory Services SNCO, Yerevan, Armenia; 9 Virology Department, Faculty of Veterinary Medicine, Ankara University, Ankara, Turkey; 10 Virology Department, Scientific Institute of Veterinary Medicine of Serbia, Belgrade, Serbia; 11 Virology Department, Institute of Veterinary Research of Tunisia, Tunis, Tunisia; 12 Laboratoire Central Vétérinaire d’Alger, Institut National de la Médecine Vétérinaire, Algiers, Algeria; 13 Animal Health Research Institute, Dokki, Egypt; 14 Department of Veterinary Medicine, Faculty of Agriculture and Veterinary Sciences, University of Prishtina, "Hasan Pristhina”, Kosovo; 15 Laboratory of Virology and Molecular Biology, LEPL State Laboratory of Agriculture (SLA), Tbilisi, Georgia; 16 Department of Animal Health, Food Safety and Veterinary Institute, Tirana, Albania; 17 Istituto Zooprofilattico Sperimentale dell'Abruzzo e del Molise "G. Caporale", Teramo, Italy; 18 CIBER Epidemiología y Salud Pública (CIBERESP), Madrid, Spain; University of Texas Medical Branch at Galveston, UNITED STATES

## Abstract

Rift Valley fever (RVF) is an arboviral zoonosis that primarily affects ruminants but can also cause illness in humans. The increasing impact of RVF in Africa and Middle East and the risk of expansion to other areas such as Europe, where competent mosquitos are already established, require the implementation of efficient surveillance programs in animal populations. For that, it is pivotal to regularly assess the performance of existing diagnostic tests and to evaluate the capacity of veterinary labs of endemic and non-endemic countries to detect the infection in an accurate and timely manner. In this context, the animal virology network of the MediLabSecure project organized between October 2016 and March 2017 an external quality assessment (EQA) to evaluate the RVF diagnostic capacities of beneficiary veterinary labs. This EQA was conceived as the last step of a training curriculum that included 2 diagnostic workshops that were organized by INIA-CISA (Spain) in 2015 and 2016. Seventeen veterinary diagnostic labs from 17 countries in the Mediterranean and Black Sea regions participated in this EQA. The exercise consisted of two panels of samples for molecular and serological detection of the virus. The laboratories were also provided with positive controls and all the kits and reagents necessary to perform the recommended diagnostic techniques. All the labs were able to apply the different protocols and to provide the results on time. The performance was good in the molecular panel with 70.6% of participants reporting 100% correct results, and excellent in the serological panel with 100% correct results reported by 94.1% of the labs. This EQA provided a good overview of the RVFV diagnostic capacities of the involved labs and demonstrated that most of them were able to correctly identify the virus genome and antibodies in different animal samples.

## Introduction

Rift Valley fever (RVF) is a zoonotic viral disease that primarily affects animals (mainly ruminants and camels), but also humans. The causative agent of RVF is an arbovirus belonging to the *Phlebovirus* genus in the *Phenuiviridae* family that was first identified in the Rift Valley of Kenya in 1930 [1]. It is transmitted to animals by mosquitos of the genus *Aedes* and *Culex* [2]. Although humans can also be infected by mosquito bites, the main route of transmission is the contact with body fluids and tissues of infected animals or the consumption of contaminated animal products such as raw or unpasteurised milk [3].

The clinical manifestations of the disease in animals vary depending on age and the species affected. Young animals are significantly more likely to succumb than adults. Sheep and goats are highly susceptible while adult cattle and camels are usually asymptomatic. In most cases the animals exhibit fever, lethargy, hepatic and splenic lesions, bloody diarrhoea and abortions. Severe disease can occur suddenly causing death without previous symptoms [2, 4].

In humans the disease is usually less severe, and the most common form is a self-limiting, flu-like illness. However, 2–5% of infected individuals may develop severe forms of the disease with haemorrhagic fever, encephalitis, retinitis, renal failure and miscarriage [5, 6]. Overall fatality rates that were historically low (around 2%) have dramatically increased (up to 40%) in the most recent outbreaks [7–9].

RVF is widespread in Africa, with spill-over to the Comoros Archipelago (including Mayotte), Madagascar, Saudi Arabia and Yemen [10]. Until now, active circulation has not been detected in Europe but RVFV introduction is a real concern due to the presence of competent vectors [11].

RVF occurs in periodic epidemics that are associated with climatic, hydrologic and socioeconomic factors. Rainfall variability is the key parameter controlling the dynamics of mosquito vectors and thereby influencing the transmission of the virus [12]. Outbreaks typically occur after unusual heavy rainfalls that provide a perfect environment for infected mosquito eggs to hatch in flooded areas [7].

The disease can have an enormous impact on the health of people and livestock and on the socio-economic outcomes. For example, in Egypt in 1976, 200.000 human cases and 600 deaths were reported [13]. In Sudan the disease caused in 2007 and 2008 747 human cases with 230 deaths [14]. The outbreaks in Somalia and Kenya in 2006–2007 were estimated to have cost 471 and 66 million US Dollars, respectively, due to livestock death, closure of livestock markets and reduced sales of animal products [15, 16]. Yemen and Saudi Arabia also suffered dire economic losses (5 and 107 million dollars, respectively) during the outbreaks in 2000–2001 [16].

RVF outbreaks always represent a great challenge for human and animal diagnostic laboratories, healthcare systems and public health institutions, as has been recently recognized in the first outbreak in Niger [8].

Human epidemics are often preceded by epizootics in livestock. Hence, surveillance of RVF in animals can act as an early warning system to prevent spill-over to humans [2, 3]. Moreover, RVF is a notifiable disease to the World Organization of Animal Health (OIE) due to the sanitary and economic consequences derived from its emergence in a free country. Although there are some commercial vaccines available (inactivated and live attenuated) for livestock in endemic countries, no vaccine for human use has yet been authorized, and the development of more reliable and safe vaccines remains a One Health challenge [17, 18]. Therefore, the implementation of surveillance systems in animals and mosquitos and the development of the diagnostic capacities of the veterinary laboratories is essential in the fight against the disease.

Diagnostic methods for RVFV detection include virus isolation, reverse-transcription polymerase chain reaction (RT-PCR) and serological tests. Isolation procedures are time-consuming and require high biocontainment facilities (BSL-3). By contrast, molecular methods, such as RT-PCR can be easily applied in basic laboratories, are fast and sensitive, allowing timely detection of the pathogen and early outbreak response. Among the serological diagnostic tools, ELISA tests are the most widely used with several kits commercially available. Virus neutralization is the OIE prescribed test for international livestock trade but it requires the use of live virus and is not recommended for use in laboratories outside endemic areas without appropriate biosecurity facilities.

MediLabSecure is an EU-funded project whose main objective is to create a framework for collaboration to promote arbovirus surveillance under a One Health approach (animal, human and entomological) in 19 countries of the Mediterranean and Black Sea regions [19–21]. The beneficiary countries recognized RVF as a common health priority, due to the devastating effects it can have in human and animal health and its potential to emerge beyond its usual geographic range. As part of the capacity building activities promoted by MediLabSecure, in 2015 and 2016 specific workshops were organized at INIA-CISA (Madrid, Spain) to improve the RVFV diagnostic capacities of the veterinary laboratories of the network. After these training sessions, an external quality assessment (EQA) was organized between October 2016 and March 2017 to evaluate the capacity of the participating labs to incorporate the acquired molecular and serological diagnostic techniques, and to promote their implementation for routine use in surveillance programs.

This study reports the results of the inter-laboratory trial and provides relevant information about the current RVFV diagnostic capacities of veterinary labs in the Mediterranean and Black Sea regions. Besides, this exercise enabled an extensive reproducibility assessment of the recommended tests for RVFV diagnostics.

## Materials and methods

### Call for participation

An invitation letter was sent by the coordinating team of the MediLabSecure animal virology network (INIA-CISA, Madrid, Spain) in May 2016 to all the involved veterinary laboratories (n = 18). Seventeen laboratories accepted to participate (94.4%). The participation was free of charge and entailed the publication of comparative results in an anonymous manner.

### Preparation of EQA panel

#### Samples for virus genome detection

For the molecular diagnosis of RVFV, each participant received a blind coded panel of 10 samples (8 positive and 2 negative), as shown in Table 1.

**Table 1:** Results of the RVFV genome detection EQA.

Strain		*MP12 vaccine*						*Namibia 2010*		*Negative samples*			
Matrix		Serum	Blood	Spleen		Liver		Spleen	Liver	Spleen	Liver		
Dilution		10^−4^	1/3 10^−4^	10^−3^	10^−2^	10^−3^	10^−2^	10^−2^	10^−2^				
Sample ID		R7	R4	R10	R5	R1	R8	R6	R2	R9	R3		
Reference Ct value		32.71 ± 0.49	31.66 ± 1.01	30.37 ± 0.70	27.07 ± 0.78	28.08 ± 0.38	24.63 ± 0.33	32.45 ± 0.80	30.19 ± 0.27	No Ct	No Ct		
**LABORATORY**	**Thermo cycler**^a^											% correct results (by lab)	Kappa value (CI 95%)
**1**	A	33.48	33.91	32.07	28.19	30.52	25.47	32.42	32.6	No Ct	No Ct	100	1 (1, 1)
**2**	B	31.58	33.03	30.58	26.96	27.95	24.57	32.35	30.97	No Ct	No Ct	100	1 (1, 1)
**3**	C	31.35	31.68	27.58	25.81	25.33	24.96	30.69	31.39	No Ct	No Ct	100	1 (1, 1)
**4**	C	31.5	29.68	28.09	25.24	26.3	24.35	30.02	28.77	No Ct	No Ct	100	1 (1, 1)
**5**	D	30.64	30.53	31.81	29.4	30.2	28.12	34.23	34.35	No Ct	No Ct	100	1 (1, 1)
**6**	B	28.5	29.2	26.5	23.2	25.6	20.5	28.7	28.2	36.9	No Ct	90	0.62 (-0.05, 1)
**7**	C	31.76	40.58^b^	29.86	25.72	26.49	24.13	31.73	30.52	No Ct	No Ct	90	0.73 (0.27, 1)
**8**	E	34.8	34.3	31.9	29.9	30.9	29.5	34.9	33.9	No Ct	No Ct	100	1 (1, 1)
**9**	B	32.9	32.23	32.6	28	28.6	25	33.3	31.16	No Ct	No Ct	100	1 (1, 1)
**10**	B	28.78	34.1	31.38	27.52	30.76	24.96	32.51	32.16	No Ct	*NA*	100	1 (1, 1)
**11**	F	33.9	31.9	32.9	29.9	29.9	26.9	32.9	31.9	No Ct	No Ct	100	1 (1, 1)
**12**	G	23.11	23.24	21.61	19.64	19.05	18.36	23.58	22.77	No Ct	No Ct	100	1 (1, 1)
**13**	G	31.89	33.08	31.5	29.45	29.52	27.56	36.58	33.01	No Ct	No Ct	100	1 (1, 1)
**14**	E	No Ct	34.5	No Ct	28.7	29.5	27.18	33.8	34	No Ct	No Ct	80	0.55 (0.05, 1)
**15**	C	26.72	30.1	26.64	23.19	25.08	21.58	28.89	27.38	No Ct	No Ct	100	1 (1, 1)
**16**	C	30.8	28.8	37	32.8	37.1	29.9	No Ct	No Ct	No Ct	No Ct	80	0.55 (0.05, 1)
**17**	C	No Ct	No Ct	No Ct	31	No Ct	33	No Ct	No Ct	30	No Ct	30	-0.129 (-0.55, 0.29)
% of correct results (by sample)		88.2	88.2	88.2	100	94.1	100	88.2	88.2	88.2	100		
CV(%)^c^		13.03	12.30	15.05	11.97	16.20	13.90	12.23	13.05	-	-		

Only results from the recommended method are shown

^a^Thermocycler used: A: Bioer GeneMax; B: Applied Biosystems 7500; C: Rotor-gene 3000, Qiagen; D: Aria Mx, Agilent; E: StepOnePlus, Applied Biosystems; F: MX 3005P, Stratagene; G: Applied Biosystems 7300

^b^Ct values above 40 are considered negative.

^c^For coefficient of variation (CV) calculations Ct values >40 and No Ct values were considered as Ct = 40

Blue: false negative results; Red: false positive result. NA: not analysed because the vial was broken upon arrival to the lab

Two viral strains were used for the preparation of the panel: Namibia 2010 [22] and the MP12 vaccine strain (ZH548-MP12-G2-P1) [23]. Both viral stocks were inactivated using ß-propiolactone. Absence of residual infectivity was confirmed after three consecutive passages in Vero cells.

Several dilutions of inactivated virus stock were spiked in different matrices (serum, blood, liver or spleen homogenates) from healthy non-infected sheep to mimic positive field samples. Liver and spleen homogenates from healthy sheep were included as negative samples. Nucleic acid extraction was performed from 200 μl of sample using the QIAamp® Cador Pathogen Mini Kit (QIAGEN), following the manufacturer’s protocol. In the final step, RNA was eluted in 50 μl of nuclease-free water. All samples were tested in duplicates by real-time RT-PCR (RRT-PCR) for RVFV detection using primers and probe described by Drosten et al. [24, 25]. Briefly, the RRT-PCR mix was prepared in a final volume of 25 μl per sample containing 5 μl of RNA template, 1 μM of each primer (RVS and RVAs), 0.2 μM of probe (RVP), RT-PCR enzyme mix and RT-PCR buffer of the commercial AgPath-ID one-step RT-PCR kit (Life Technologies, Thermo Fisher Scientific). All reactions were carried out in a Mx3005P equipment with the following thermal profile: reverse transcription at 45°C for 10 min, initial PCR activation step at 95°C for 10 min, followed by 45 cycles of 15 sec at 95°C, 20 sec at 56°C, and 30 sec at 72°C. According to the obtained Ct values, a collection of 10 samples was finally selected (Table 1). The samples were aliquoted (1 ml) and each vial was lyophilized and stored at 4°C until delivery to the participant laboratories. Prior to delivery, the lyophilized panel was resuspended in DNAse free water and was fully analysed to verify the integrity of the samples. Triplicates of each lyophilised sample were analysed by RRT-PCR at INIA-CISA by 3 technicians. The reference Ct value was established as the mean of the nine repetitions (Table 1). The within-laboratory repeatability of the assay was evaluated for the positive samples, obtaining a coefficient of variation (CV) range of 0.89–3.19% (mean 1.99%).

Two positive controls were delivered with the panel: (1) a positive nucleic acid extraction control consisting of cell culture medium spiked with inactivated RVFV MP12 vaccine strain to obtain, after a 1/10 dilution, an expected Ct value of 32±2 (this sample was lyophilized and stored at 4°C until delivery) and (2) a positive reaction control consisting of RVFV positive RNA with an expected Ct value of 32±2 (this sample was stored at -80°C until delivery).

#### Samples for antibody detection

For the serological diagnosis of RVFV, each participant received a blind coded panel of 10 sheep sera (6 positive and 4 negative). The positive samples consisted of sera from 3 sheep vaccinated with the formalin-inactivated RVFV vaccine from Onderstepoort Biological Products (OBP, Onderstepoort, South Africa). The sera were inactivated by heating at 56°C for 2 hours and then analysed by ELISA (ID Screen Rift Valley Fever Competition Multi-species, IDVET) and virus neutralization test using the MP12 vaccine strain (ZH548-MP12-G2-P1). According to the obtained results, several dilutions were selected to prepare the positive samples of the panel (Table 2). The negative samples consisted of sera from healthy cattle. Each sample was aliquoted (130 μl) and stored at -20°C until shipment. The reference quantitative OD values of the panel were established at INIA-CISA by repeating the assay 3 times by three different technicians. The within-laboratory repeatability, estimated as CV of the OD values obtained, ranged over 3.87–23.66% (mean 14.68%).

**Table 2:** Results of the RVFV antibody detection EQA.

	R6	R3	R4	R9	R10	R1	R2	R7	R8	R5		
Serum sample	*Vaccinated sheep (#1)*	*Vaccinated sheep (#1)*	*Vaccinated sheep (#2)*	*Vaccinated sheep (#2)*	*Vaccinated sheep (#3)*	*Vaccinated sheep (#3)*	*Healthy cattle*	*Healthy cattle*	*Healthy cattle*	*Healthy cattle*		
Dilution	1:4	1:15	1:8	1:12	1:10	1:15						
Qualitative result	+	+	+	+	+	+	-	-	-	-		
**LABORATORY**											% of correct results (by lab)	Kappa value (CI 95%)
**1**	+	+	+	+	+	+	-	-	-	-	100	1 (1, 1)
**2**	+	+	+	+	+	+	-	-	-	-	100	1 (1, 1)
**3**	+	+	+	Doubtful	+	+	-	-	-	-	90	0.82 (0.50, 1)
**4**	+	+	+	+	+	+	-	-	-	-	100	1 (1, 1)
**5**	+	+	+	+	+	+	-	-	-	-	100	1 (1, 1)
**6**	+	+	+	+	+	+	-	-	-	-	100	1 (1, 1)
**7**	+	+	+	+	+	+	-	-	-	-	100	1 (1, 1)
**8**	+	+	+	+	+	+	-	-	-	-	100	1 (1, 1)
**9**	+	+	+	+	+	+	-	-	-	-	100	1 (1, 1)
**10**	+	+	+	+	+	+	-	-	-	-	100	1 (1, 1)
**11**	+	+	+	+	+	+	-	-	-	-	100	1 (1, 1)
**12**	+	+	+	+	+	+	-	-	-	-	100	1 (1, 1)
**13**	+	+	+	+	+	+	-	-	-	-	100	1 (1, 1)
**14**	+	+	+	+	+	+	-	-	-	-	100	1 (1, 1)
**15**	+	+	+	+	+	+	-	-	-	-	100	1 (1, 1)
**16**	+	+	+	+	+	+	-	-	-	-	100	1 (1, 1)
**17**	+	+	+	+	+	+	-	-	-	-	100	1 (1, 1)
% of correct results (by sample)	100	100	100	94.1	100	100	100	100	100	100		

Only results from the recommended method are shown.

Blue: incorrect result

### EQA details

This exercise was conceived as a follow-up of the diagnostic training workshops that the beneficiary labs attended at INIA-CISA in 2015 and 2016. The main objective was to evaluate the capacity of the labs to perform the diagnosis of RVFV using the protocols, kits and reagents that were used during the training sessions.

For the molecular detection of RVFV we selected the OIE recommended RRT-PCR protocol [25] using the primers and probe described by Drosten et al. [24] targeting the RVFV-M segment.

For serological diagnosis of the infection, we recommended the use of the ID Screen Rift Valley Fever Competition Multi-species (IDVet) ELISA, a commercially available kit that has been widely used in surveillance programs, with optimal sensitivity and specificity [26, 27].

Detailed standard operating procedures were distributed and all the reagents and kits were provided to each laboratory including the nucleic acid extraction kit (QIAamp® Cador Pathogen Mini Kit, QIAGEN), the RT-PCR kit (AgPath-ID one-step RT-PCR kit, Life Technologies, Thermo Fisher Scientific), primers and probe [24] and the mentioned IDVet ELISA kit. As explained before, extraction and reaction positive controls were also delivered to all the labs.

For the molecular panel, specific instructions for the reconstitution of the lyophilized samples as well as for preparation of the positive extraction control were provided to all the labs. For the serological panel, the participant laboratories were asked to analyse the panel samples using the provided commercial kit following manufacturer’s instructions.

Additionally, it was suggested to analyse both panels using alternative methods (other protocols that may be established in the labs) and report the results together with those derived from the recommended methods.

The lyophilized samples, the positive extraction control and the nucleic acid extraction and ELISA kits were shipped at room temperature. The panel of sera, the RT-PCR kit, the positive reaction control and the primers and probe were shipped in dry ice.

A number code was assigned to each laboratory to ensure a blind analysis of the results.

### Ethics statement

The tissue samples and sera used for the preparation of the EQA panels were selected from the biobanks of INIA-CISA (Madrid, Spain) and IZSAM (Teramo, Italy). None of them were specifically collected for this study. Positive sheep sera were obtained from a vaccination study that was conducted by IZSLER in accordance with Italian law for animal protection and with the European Directive 2010/63/EU. The experiment was approved by the Internal Animal Welfare body and authorized by the local authorities (n. 998 22/09/2015 Italian Minister of Health). The organs and blood used to prepare the rest of the panel samples were obtained from the biobank of INIA-CISA. All of them originated from animal studies that were authorized by INIA Animal Experimentation Ethics Committee according to European Directive 2010/63/EU (Spanish Royal Decree 53/2013).

## Results

**Fig 1:**
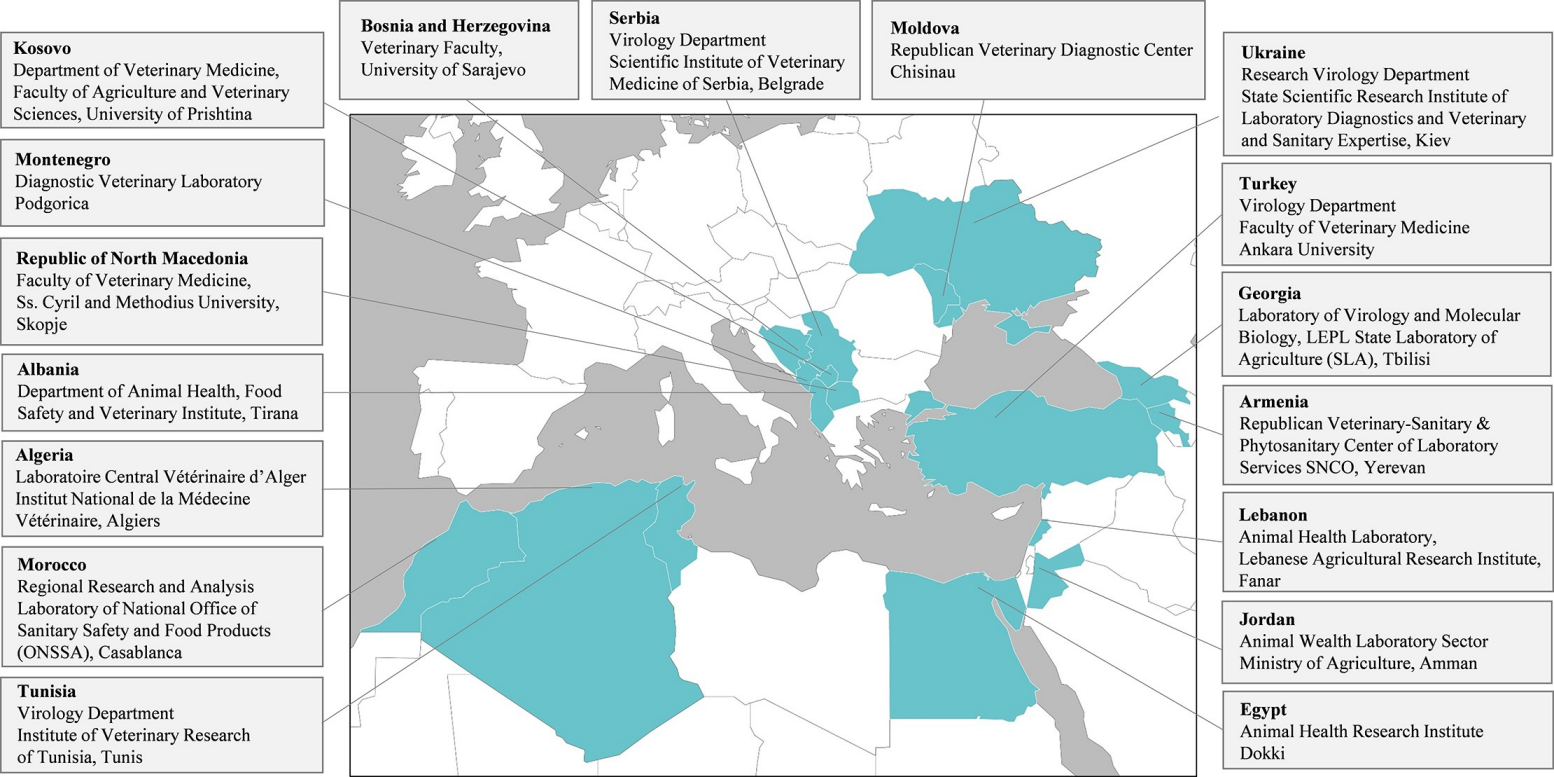
Participant laboratories.

Seventeen laboratories submitted results, representing 17 countries from the Mediterranean and Black Sea regions (Fig 1).

### Virus genome detection

**Fig 2:**
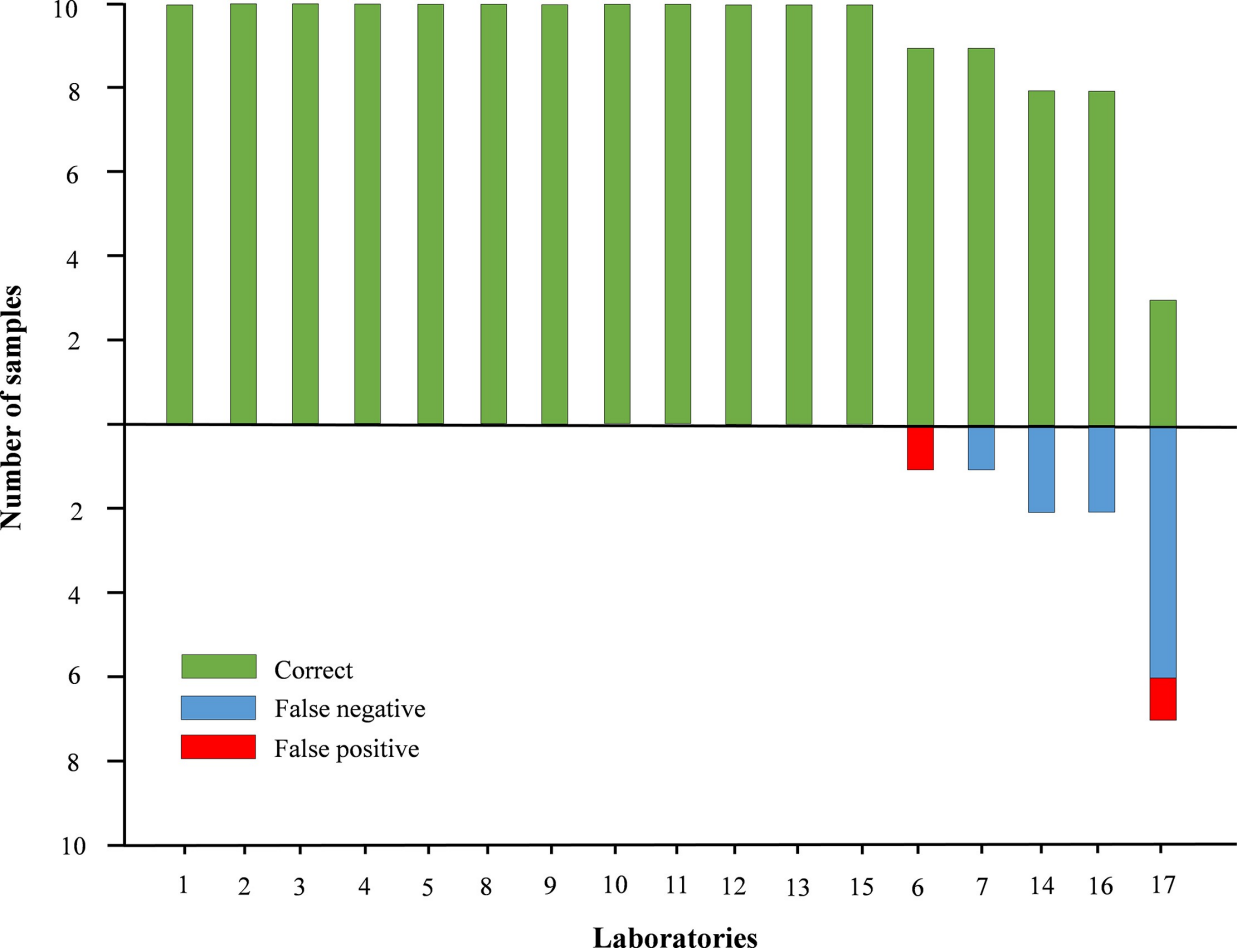
RVFV genome detection EQA performance of individual laboratories. Bars above the baseline indicate correct results; bars below the baseline indicate incorrect results.

A total of 18 datasets were received from the 17 labs, including one double set from laboratory #1 that used an alternative method apart from the recommended one. The results of all the labs using the recommended method are shown in Table 1 and Fig 2.

The labs reported the use of seven different thermocyclers: Rotor-Gene 3000 (6 labs), Applied Biosystems 7500 (4 labs), Applied Biosystems 7300 (2 labs), StepOnePlus (2 labs), Aria Mx Agilent (1 lab), Bioer Gene Max (1 lab) and Stratagene Mx3005 (1 lab).

Twelve out of 17 laboratories (70.6%) reported 100% of correct results using the recommended protocols and reagents. The laboratory providing two different RT-PCR datasets produced 100% of correct results with the recommended protocol and 3 false negative results (R6, R7 and R8) with the commercial kit RVFV dtec-RT-qPCR Test, GPS Genetic PCR Solutions.

In general, the reported Ct values were in line with the reference values. However, three labs (#5, #8 and #13) reported Ct values slightly higher (2–4 Cts) than expected in some samples and 2 labs (#6 and #15) reported Ct values slightly lower (2–4 Cts) than expected. It is noteworthy that in one lab (#12) the reported Ct values were considerably lower (6–9 Cts) than the reference values.

Only one lab (#17) reported more than two incorrect results. In fact, this lab misidentified 70% of the samples and the reported Ct values were higher than expected.

The percentage of laboratories with correct results increased as the expected Ct value of the sample decreased (higher viral load), as shown in Table 1. Samples R5 and R8 with the lowest Ct values were correctly assigned by all the labs.

The reproducibility of the assay in terms of qualitative results was excellent (kappa = 1) for the twelve laboratories with 100% of correct results, substantial for 2 laboratories that failed in 1 sample (#6 and #7, kappa values of 0.62 and 0.73, respectively) and moderate for 1 laboratory that failed in 2 samples (#16, kappa = 0.55) (Table 1).

The reproducibility of the assay for the positive samples, in terms of quantitative values (Ct) obtained, expressed as CV, ranged from 11.97 to 16.20% (mean: 13.47%; Table 1).

### Antibody detection

A total of 18 datasets were received from the 17 labs, including one double set from laboratory #1 that used an alternative ELISA kit apart from the recommended one. The results of all the labs with the recommended method are shown in Table 2.

Sixteen out of 17 labs (94.1%) reported correct results for all the samples using the recommended ELISA method showing an excellent reproducibility between laboratories (kappa value = 1). Only one laboratory (#3) obtained one incorrect result (sample R9 was reported as doubtful instead of positive), reaching also a high reproducibility (kappa value = 0.82). The laboratory providing two different datasets produced 100% of correct results with the recommended kit and four false negative results (R1, R4, R9 and R10) with the alternative kit (Ingezim FVR Compac, Ingenasa).

## Discussion

Since the beginning of the MediLabSecure project in 2014, the animal virology network has implemented numerous actions to strengthen awareness, diagnosis and control of emerging vector-borne viruses of great impact in human and animal health. As part of the capacity building activities, two workshops were organized in 2015 and 2016 to provide specialized training in molecular and serological diagnosis of the arboviruses that were identified as major health concerns in the region, namely West Nile virus and RVFV.

A further step of this training curriculum was the organization in 2016–2017 of two external quality assessments to evaluate the acquired capacities of each laboratory to detect both viruses from animal samples. In this article we present the results of the RVFV inter-laboratory assay.

Despite the increasing incidence of RVFV in Africa with recent and severe outbreaks in new countries such as Niger [8] and continuous spill-over to other regions as Saudi Arabia and Comoro Islands [28], very few external quality assessments have been implemented to evaluate the capacity of the labs to accurately detect RVFV genome and antibodies in clinical samples and to harmonize the diagnostic techniques. In the human virology sector, one EQA was organized by the ENIVD network in 2012 to evaluate the molecular diagnostic capacities of 30 labs of 16 countries [29]. In the veterinary sector, only one EQA was organized in 2013–2014 by IZSAM in collaboration with WHO and FAO in the framework of the REMESA project [27]. Ten laboratories in six countries from Europe and North Africa participated to assess their molecular and serological diagnostic performances.

The EQA we present here has a number of particular features that differentiate it from previous RVFV EQAs. In the first place, the number and geographical coverage of the involved laboratories. In this case, 17 veterinary laboratories from 17 non-EU countries have participated including North Africa, Balkans, Black Sea and Middle East regions. Of these countries only 3 labs in Morocco, Tunisia and Algeria had participated in the previous EQA organized by IZSAM [27]. Secondly, this EQA was designed as the last step of a 3-years training curriculum to evaluate the capacity of the trainees to incorporate the techniques acquired during the workshops into their own laboratories. Last, but not least, the labs were provided with all the reagents and kits required to perform the diagnostic assays following the standard operating procedures that were distributed during the workshops. The objective was to avoid difficulties of the labs to purchase/obtain the requested diagnostic materials and facilitate as much as possible the participation of all beneficiary institutions. Moreover, and as part of the preparedness actions promoted by MediLabSecure, positive nucleic acid extraction and reaction controls were also provided to be used as quality controls during this EQA but also to serve as reference material for future diagnostic activities of the labs. Such material is otherwise difficult to obtain.

The combination of molecular and serological methods in the same EQA is also an added value, as the surveillance of RVFV in animals requires this complementary approach, particularly in non-endemic countries.

The panel for RVFV genome detection consisted of 10 samples containing various concentrations of two RVFV strains. Apart from the well characterized strain Namibia 2010 that was used in the previously mentioned EQA by Monaco and colleagues [27], we included an additional strain, the MP12 vaccine strain, that has been used as live attenuated vaccine in animals and has been proposed as vaccine candidate for humans [18, 23].

In previous EQAs, the viruses were diluted in human plasma [29] or bovine serum [27], but in this case, and to mimic as much as possible the clinical samples that the vet labs would receive during surveillance and outbreak investigations, the two viruses were spiked in different target organs/matrices (serum, blood, liver and spleen).

The panel for antibody detection consisted of six sera from vaccinated sheep with different antibody titres and four sera from healthy cattle. Altogether, we consider that both panels represent a comprehensive proficiency test to efficiently evaluate the actual RVFV diagnostic capacities of the labs.

The performance in the molecular panel was good in general terms, as 70.6% of participants reported 100% correct results. Surprisingly, one laboratory reported very poor results, with one false positive and six false negative results. It seems that a technical error could occur during the extraction process obtaining a significantly lower RNA yield in the whole panel. In fact, and as part of the corrective measures implemented after the analysis of EQA results, a new panel of samples was shipped to this laboratory obtaining 100% concordant results. So, if we discard the results of this laboratory, the percentage of labs with 100% correct results would increase to 75%. Only one lab reported a false positive result and three labs reported one, two and two false negative results, respectively.

Overall, the reported Ct values were concordant with the reference values, although some variability (ranging from 1 to 4 Ct units) was observed in certain laboratories, which could be due to the different thermocyclers used and/or to minor performance variations in the entire analysis process. Only one laboratory (#12) reported very discrepant Ct values in the whole panel, with a deviation of 6–9 Ct units lower than the reference data. Considering that all the labs used exactly the same protocols, reagents and kits, the only lab-specific element was the thermocycler, and although differences in calibration or in threshold setting could contribute to certain Ct variation, it can hardly account for such a big difference in RT-PCR performance as compared with the rest of participants. Moreover, another lab used the same equipment (Applied Biosystems 7300) and obtained Ct values concordant with the reference data.

Only one laboratory (#1) reported the results of an additional RRT-PCR method using a commercial kit (RVFV dtec-RT-qPCR Test) purchased from “GPS Genetic PCR Solutions” company. In this case, three positive samples (two with expected Ct>32 and one with expected Ct<25) were wrongly assigned as negative. Since only one lab used this commercial kit, the apparent low sensitivity of this method should be cautiously interpreted.

It is worth noting that the equipment and expertise necessary to apply RRT-PCR techniques was available in all the labs, which confirms that the use of RRT-PCR has substantially increased and is nowadays the primary molecular diagnostic method in the involved veterinary labs.

The results of the antibody detection EQA were excellent, with 100% of correct results in all labs except for one positive sample that lab #3 reported as doubtful. Competition values (not shown) obtained in all the labs were concordant with the reference values. The analysis of the panel with an additional ELISA kit (Ingezim FVR Compac Ingenasa), used by laboratory #1, yielded 40% of incorrect results (false negatives). However, this data should be interpreted with precaution as only one laboratory used the kit.

The unique characteristics of this EQA, in which all the protocols and technical components (except the thermocycler and ELISA reader) are the same in all participant labs allows to accurately evaluate the reproducibility of the selected diagnostic methods. In this way, we could verify that the RRT-PCR protocol designed by Drosten et al. [24] is a reliable method for accurate detection of RVFV genome. The use of this protocol in previous EQAs in human and animal samples also yielded very good results [27, 29] and it has also been successfully used to identify the presence of the viral genome in clinical samples during epidemiological investigations [10].

In the case of the antibody detection exercise, our results confirm optimal performance of the IDVet kit, with high reproducibility values, as all the labs reported 100% concordant results (except one positive serum identified as doubtful). This commercial kit is widely used for RVFV diagnosis in ruminants as demonstrated in the EQA conducted by IZSAM, where all the labs selected this kit for the analysis of the sera [27]. Furthermore, this ELISA displayed the best analytical sensitivity and specificity values in a ring trial organized to evaluate the performance of different ELISAs for the diagnosis of RVFV infections [26].

In conclusion, this exercise provides a good overview of the RVFV diagnostic capacities of veterinary labs in 17 EU-neighbouring countries. Most of the participant labs demonstrated their ability to correctly identify RVFV genome and antibodies in different animal samples. All the labs were able to apply the recommended diagnostic protocols and to provide the results on time. Notably, before the implementation of this MediLabSecure diagnostic training program in 2015, only 37.5% of the beneficiary labs had established protocols to diagnose RVFV infections. The proposed training strategy with two workshops (molecular and serological diagnosis) followed by this EQA has proved very useful in upgrading the capacities of the labs. However, regular refresher trainings and EQA programs should be encouraged to maintain and improve this expertise.
